# Serum Parathyroid Hormone, 25-Hydroxyvitamin D, and Risk of Alzheimer’s Disease: A Mendelian Randomization Study

**DOI:** 10.3390/nu10091243

**Published:** 2018-09-06

**Authors:** Susanna C. Larsson, Matthew Traylor, Hugh S. Markus, Karl Michaëlsson

**Affiliations:** 1 Unit of Nutritional Epidemiology, Institute of Environmental Medicine, Karolinska Institutet, SE-171 77 Stockholm, Sweden; 2 Stroke Research Group, Department of Clinical Neurosciences, University of Cambridge, Cambridge 01223, UK; mt628@medschl.cam.ac.uk (M.T.); hsm32@medschl.cam.ac.uk (H.S.M.); 3 Department of Surgical Sciences, Uppsala University, 75105 Uppsala, Sweden; karl.michaelsson@surgsci.uu.se

**Keywords:** Alzheimer’s disease, Mendelian randomization, parathyroid hormone, vitamin D

## Abstract

We conducted Mendelian randomization analyses to investigate the associations of serum parathyroid hormone (S-PTH) and serum 25-hydroxyvitamin D (S-25OHD) concentrations with Alzheimer’s disease (AD). Five and seven single nucleotide polymorphisms associated with S-PTH and S-25OHD concentrations, respectively, were used as instrumental variables. Data for AD were acquired from the International Genomics of Alzheimer’s Project (17,008 AD cases and 37,154 controls). Genetically higher S-PTH concentrations were not associated with AD (odds ratio per standard deviation increase in S-PTH = 1.11; 95% CI 0.97–1.26; *p* = 0.12). In contrast, all seven 25OHD-increasing alleles were inversely associated with AD and two of the associations were statistically significant (*p* < 0.05). The odds ratio of AD per genetically-predicted one standard deviation increase in S-25OHD was 0.86 (95% CI 0.78–0.94; *p* = 0.002). This study provides evidence that vitamin D may play a role in AD but found no significant association between S-PTH and AD.

## 1. Introduction

Accumulating evidence from experimental [1,2,3,4,5] and observational epidemiological [6,7,8,9,10] studies indicates that vitamin D may play a role in Alzheimer’s disease (AD). Moreover, Mendelian randomization studies have shown a suggestive inverse association between genetically predicted serum 25-hydroxyvitamin D (S-25OHD) concentrations, based on four single-nucleotide polymorphisms (SNPs), and AD [11,12]. Both vitamin D receptors and 1α-hydroxylase, the enzyme responsible for the formation of the biologically active form of vitamin D (1, 25-dihydroxyvitamin D), are present in the hippocampus [13]. The activity of 1a-hydroxylase is stimulated by parathyroid hormone (PTH), and receptors for this hormone are also found in the brain [14]. Elevated serum PTH (S-PTH) concentrations have been linked to poor cognition, vascular dementia, and all-cause dementia in some studies [15,16], but whether S-PTH concentrations affect the risk of AD specifically is unclear. S-PTH and S-25OHD are inversely interrelated [17] and there is a need to further disentangle their effects on AD.

To provide insights into the role of PTH in AD, we performed a Mendelian randomization study to genetically predict the association of S-PTH concentrations with AD using data for five SNPs explaining 4.2% of the variance in S-PTH concentrations [18]. We also conducted an updated Mendelian randomization analysis of vitamin D and AD by using data for seven SNPs explaining about 7.5% of the variance in S-25OHD concentrations [19,20].

## 2. Methods

### 2.1. Study Design and Data Sources

We conducted a two-sample Mendelian randomization study using SNPs associated with S-PTH or S-25OHD as instrumental variables. We selected the SNPs associated with S-PTH or S-25OHD concentrations at genome-wide significance (*p* < 5 × 10^−8^) in previously published genome-wide association studies of respectively 29,155 [18] and 79,366 [19] individuals of European ancestry (Table 1). None of the SNPs within each trait were in linkage disequilibrium (*r*^2^ < 0.05). In addition, for S-25OHD concentrations, we included the low-frequency SNP (rs10741657) of large effect on S-25OHD that was described in a genome-wide association study of 42,274 individuals of European ancestry (Table 1) [20]. This variant in *CYP2R1* is not in linkage disequilibrium with the other common *CYP2R1* variant (*r*^2^ between the two SNPs = 0.03) (Table 1) [20].

Summary-level data (β-coefficients and standard errors) for the associations of the five S-PTH-associated SNPs and seven S-25OHD-associated SNPs with AD were available from the International Genomics of Alzheimer’s Project (IGAP), comprising 17,008 cases of AD and 37,154 controls of European ancestry [21]. All AD cases were confirmed by standardized diagnostic criteria (National Institute of Neurological and Communicative Disorders and Stroke and the Alzheimer’s Disease and Related Disorders Association (NINCDS-ADRDA); Diagnostic and Statistical Manual of Mental Disorders, 4th Edition (DSM-IV)) [21].

This study is based on publicly available summarized (i.e., aggregated) data. Individual studies within each genome-wide association study received approval from a relevant Institutional Review Board, and informed consent was obtained from participants or from a caregiver, legal guardian, or other proxy.

### 2.2. Mendelian Randomization Analyses

The inverse-variance weighted method was used in the primary analysis [22]. In sensitivity analyses, the weighted median and MR-Egger methods were used to explore pleiotropy. The Mendelian randomization estimates were scaled per one standard deviation (SD) of ln-transformed S-PTH and S-25OHD. Approximate SDs were derived from the population-based Swedish Mammography Cohort Clinical study [23] and was 0.33 ln-pg/mL for S-PTH and 0.33 ln-nmol/L for S-25OHD. Analyses were conducted using Stata (StataCorp, College Station, TX, USA).

## 3. Results

**Figure 1:**
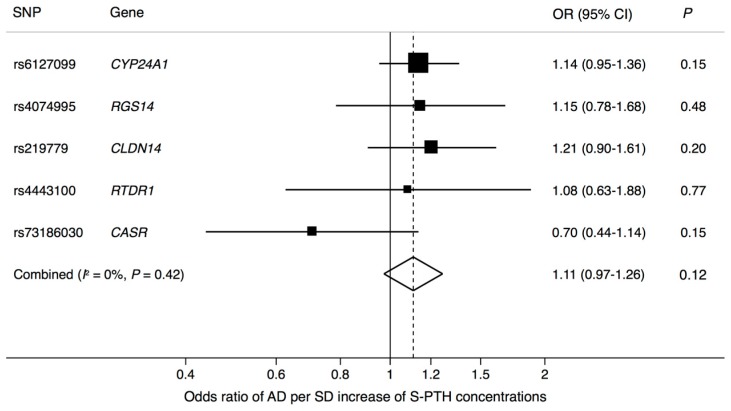
Association between a genetically-predicted one standard deviation increase of S-PTH concentration and AD.

There was no significant association between genetically higher S-PTH concentrations and AD (odds ratio (OR) = 1.11; 95% confidence interval (CI), 0.97–1.26; *p* = 0.12) (Figure 1). The lack of association remained in a sensitivity analysis using the weighted median method (OR = 1.14; 95% CI, 0.97–1.34; *p* = 0.10). There was no evidence of pleiotropy in the MR-Egger analysis (intercept: −0.013 (95% CI −0.055 to 0.029), *p* = 0.55).

**Figure 2:**
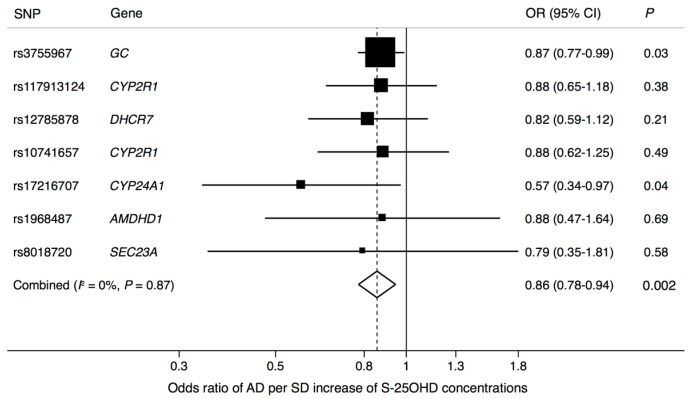
Association between a genetically predicted one standard deviation increase of S-25OHD concentration and Alzheimer’s disease. rs1968487 was used as proxy SNP for rs10745742 (*r*^2^ between these two SNPs = 1.0).

All seven S-25OHD-increasing alleles were inversely associated with AD and two of the associations were statistically significant (*p* < 0.05) (Table 1). In the Mendelian randomization analysis using information for all seven SNPs, the OR of AD per genetically-predicted one SD increase of serum S-25OHD concentration was 0.86 (95% CI 0.78–0.94; *p* = 0.002), without heterogeneity among individual SNPs (Figure 2). Results were robust in a sensitivity analysis using the weighted median method (OR = 0.87; 95% CI 0.78–0.98; *p* = 0.02). There was no evidence of pleiotropy in the MR-Egger analysis (intercept: −0.008 (95% CI −0.032 to 0.016), *p* = 0.51).

## 4. Discussion

This Mendelian randomization study provides strong evidence that higher S-25OHD concentrations are associated with a reduced risk of AD. Genetically higher S-PTH concentrations were not associated with AD, but we cannot rule out that a weak association may have been overlooked.

Previous Mendelian randomization analyses have demonstrated that four S-25OHD-associated SNPs in or near *GC, DHCR7, CYP2R1,* and *CYP24A1* and a combined genetic risk score of those genetic variants are associated with AD (*p* = 0.01) [11,12]. The present analysis extends those results by showing that three additional S-25OHD-associated SNPs are associated with AD in the same direction and of the same magnitude, and a combined score of all seven SNPs shows that each SD increment of S-25OHD concentration (about 20–30 nmol/L or 8–12 ng/ml) is associated with a 14% reduced risk of AD (*p* = 0.002).

Our results are consistent with most [6,7,8,9] but not all [24,25] observational prospective studies, which have found that low pre-diagnostic S-25OHD concentrations are associated with an increased risk of AD. The reason for the inconsistent results among observational studies could be related to small sample sizes and, thus, low power in some of the studies, as well as differences in average S-25OHD concentrations among individuals in the reference group and the group defined as having low vitamin D status. Case-control and cross-sectional studies have shown a lower mean S-25OHD concentration in AD patients than in controls [10], but reverse causality is a potential concern in such studies since AD patients may have reduced their outdoor activities or changed their diet, potentially leading to decreased S-25OHD concentrations.

Studies on vitamin D and cognitive outcomes have generally shown no association. A Mendelian randomization study found no association between a genetic risk score of SNPs in the *DHCR7* and *CYP2R1* gene regions and cognitive performance [26]. Randomized controlled trials of vitamin D supplementation on cognitive outcomes in patients suffering from AD are limited. One trial showed that treatment with vitamin D plus memantine was associated with improvement in cognition compared with vitamin D or memantine alone [27]. Two other trials failed to find an impact on vitamin D supplementation on cognitive performance in nursing home residents and individuals with mild-moderate severe AD [28,29]. A post hoc analysis of the Women’s Health Initiative, including 4143 women aged 65 years and older without probable dementia, showed no difference in incident dementia or cognitive function between women who received 400 IU of vitamin D_3_ combined with 1000 mg of calcium and women who received placebo [30]. Whether long-term vitamin D supplementation prevents the onset or progression of AD is unknown and merits evaluation in a randomized controlled trial. Such a trial may, however, not be feasible to conduct due to the large sample size and long follow-up required to detect an association.

Vitamin D affects several mechanisms of AD pathogenesis, including the production, aggregation, clearance, and degradation of amyloid β peptides, as well as neurogenesis and Tau phosphorylation [1,2,3,4,5]. For instance, experimental studies in mouse models of AD have shown that vitamin D treatment improves neurogenesis [3] and reduces amyloid b deposition, particularly in the hippocampus where the vitamin D receptor is abundant [4]. Vitamin D is also involved in the transcriptional activation of the tryptophan hydroxylase 2 gene, which encodes the rate-limiting enzyme in serotonin synthesis [31]. Serotonin may play a role in AD by influencing neurogenesis and amyloid plaque load [32].

The association between S-PTH and dementia has been examined in a few prospective and case-control studies [15,16]. In a prospective cohort of 988 Swedish men, high S-PTH concentrations were significantly positively associated with risk of vascular dementia (41% increase in risk per 1 SD increase in S-PTH) but were not associated with risk of AD or other dementias [16]. Another prospective study found that elevated S-PTH concentrations were associated with a significant increased risk of all-cause dementia during the one- and five-year follow-up, but not at the ten-year follow-up [33]. Reverse causality may explain the observed association in that study since a large proportion of participants had cognitive impairment at baseline and the association was not consistent at the ten-year follow-up. Two small case-control studies reported significantly higher S-PTH concentrations among patients with dementia [34] or AD specifically [35] compared with controls. However, case-control studies are susceptible to reverse causation bias.

A major strength of the Mendelian randomization approach is that reverse causation bias is avoided because genetic variants are fixed at conception. Another strength is that confounding is reduced by the use of genetic variants as proxies for the exposure and that long-term exposure is investigated. In addition, since there is an inverse physiological correlation between S-PTH and S-25OHD [17] their independent effects cannot easily be evaluated by a non-instrumental study design.

A limitation is potential bias due to pleiotropy, which occurs when one gene influences multiple traits. We cannot entirely exclude that the SNPs used as instruments in the present study may affect the risk of AD through mechanisms other than their effects on S-25OHD. However, findings were similar in a complementary analysis using the weighted median method, which provide robust estimates when at least 50% of the weight in the analysis derives from valid instruments. We found no evidence of directional pleiotropy in the MR-Egger analysis but this method has low power when the number of SNPs is few. Other modifiable exposures, such as cardiometabolic risk factors (e.g., body mass index, waist-to-hip ratio, blood pressure, fasting insulin, fasting glucose, and cholesterol levels), were not associated with AD in a recent Mendelian randomization study based on the IGAP dataset [12] and, therefore, are unlikely confounders of the association of S-25OH with AD. Another possible concern in Mendelian randomization studies is population stratification. Nonetheless, this potential source of bias was reduced since the genome-wide association studies on S-25OH and AD only included European-descent individuals.

## 5. Conclusions

This study indicates that genetically higher concentrations of S-25OHD, but not S-PTH, are associated with AD. This suggests that maintenance of an adequate vitamin D status throughout life may reduce the risk of AD.

## Figures and Tables

**Table 1:** Characteristics of the single nucleotide polymorphisms associated with S-PTH and S-25OHD concentrations and their associations with AD.

Exposure	SNP	Chr	Nearby Gene	EA *	EAF	Associations with Exposure			Associations with AD		
						β ^†^	SE	*p*	β ^‡^	SE	*p*
S-PTH	rs6127099	20	*CYP24A1*	T	0.34	0.07	0.003	2.4 × 10^−72^	0.028	0.019	0.155
S-PTH	rs4074995	5	*RGS14*	G	0.71	0.03	0.003	3.3 × 10^−23^	0.012	0.018	0.482
S-PTH	rs219779	21	*CLDN14*	G	0.75	0.04	0.003	8.9 × 10^−22^	0.023	0.018	0.202
S-PTH	rs4443100	22	*RTDR1*	G	0.32	0.02	0.003	4.1 × 10^−11^	0.005	0.017	0.773
S-PTH	rs73186030	3	*CASR*	T	0.14	0.03	0.004	1.2 × 10^−9^	−0.032	0.022	0.151
S-25OHD	rs3755967	4	*GC*	C	0.72	0.089	0.002	4.74 × 10^−343^	−0.036	0.017	0.034
S-25OHD	rs117913124	11	*CYP2R1*	G	0.975	0.430 ^#^	0.020	1.50 × 10^−88^	−0.057	0.065	0.378
S-25OHD	rs12785878	11	*DHCR7*	T	0.75	0.036	0.002	3.80 × 10^−62^	−0.022	0.018	0.208
S-25OHD	rs10741657	11	*CYP2R1*	A	0.40	0.031	0.002	2.05 × 10^−46^	−0.011	0.016	0.492
S-25OHD	rs17216707	20	*CYP24A1*	T	0.79	0.026	0.003	8.14 × 10^−23^	−0.045	0.022	0.038
S-25OHD	rs10745742 ^§^	12	*AMDHD1*	T	0.40	0.017	0.002	1.88 × 10^−14^	−0.006	0.016	0.691
S-25OHD	rs8018720	14	*SEC23A*	G	0.18	0.017	0.003	4.72 × 10^−9^	−0.012	0.021	0.584

Abbreviations: Chr, chromosome; EA, effect allele; EAF, effect allele frequency; SE, standard error. * Allele associated with higher concentrations of the exposure. ^†^ β coefficients of the exposure-increasing allele on natural log-transformed concentrations of S-PTH (obtained from Robinson-Cohen et al. [18]) and S-25OHD (obtained from Jiang et al. [19]), if not otherwise indicated. ^‡^ β coefficients of the exposure-increasing allele on log odds ratio of AD (obtained from Lambert et al. [21]). ^§^ SNP rs10745742 was not available in the dataset for AD. Therefore, rs1968487 was used as proxy (*r*^2^ between these two SNPs = 1.0). ^#^ β coefficient of the exposure-increasing allele on standard deviations of the standardized log-transformed S-25OHD concentrations (obtained from Manousaki et al. [20]).
